# Snocross “Shark-bite” Laceration

**DOI:** 10.5811/cpcem2022.6.57128

**Published:** 2022-08-08

**Authors:** Wyatt Telken, Jon Solberg, Mark Raymond

**Affiliations:** University of North Dakota School of Medicine and Health Sciences, Department of Emergency Medicine, Bismarck, North Dakota

**Keywords:** Snocross, laceration, shark bite, motorsport, wound

## Abstract

**Case Presentation:**

A snowmobile racer fell from his sled and was run over by another, sustaining “shark bite” to his hand and leg. He was evacuated to a trackside medical trailer where the characteristic wounds were felt to require further exploration at a hospital.

**Discussion:**

“Shark bite” is a colloquial term for lacerations sustained from metal studs attached to a snowmobile’s track. “Shark-bite” lacerations may be more prone to complications than other lacerations commonly sustained in motorsports events.

## CASE PRESENTATION

**Image 1:**
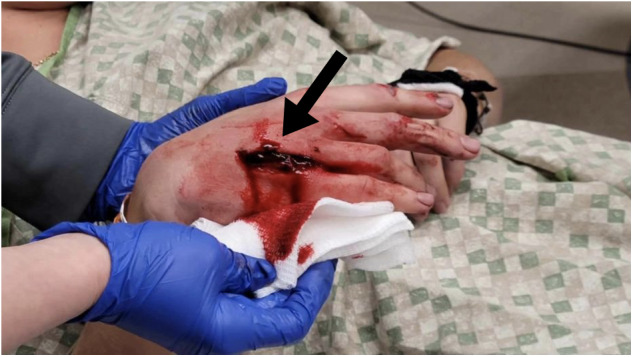
Image of the patient’s right hand on presentation to the hospital, demonstrating a laceration (arrow) to the dorsal side between the fourth and fifth metacarpals. Patient had weakness to extension of the right fingers and was found to have extensor tendon lacerations.

**Image 2:**
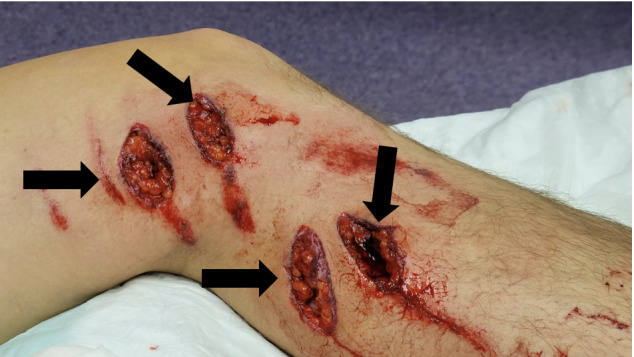
Image of the left thigh and calf demonstrating four deep lacerations as well as multiple abrasions. These lacerations (arrows) are referred to as “shark bite” in the world of Snocross competition due to their resemblance to wounds experienced by those in a shark attack. They occur when the rider is run over by the metal treads of the snowmobile track. They are powerful enough to tear through the racer’s thick winter gear to cause these deep lacerations.

A 30-year-old male Snocross (competitive snowmobile racing) rider fell from his snowmobile and was run over by another rider. An emergency medical technician who evaluated him trackside found a painful, bloody hand and ripped pants saturated with blood. The patient was able to be transported to a trackside medical trailer. Physical exam was significant for a right hand (Image 1) open laceration dorsally with fourth and fifth digits held in passive flexion with sensation intact, and left leg (Image 2) with several linear lacerations extending at least into the subcutaneous fat. The “shark bite” associated with metal cleats from a snowmobile track can be deep, contaminated, associated with significant underlying injury, and prone to tetanus. The wounds were covered with saline-soaked gauze and wrapped. The hand was placed in a volar splint of malleable aluminum and wrapped with an elastic bandage. The patient was transported to a local hospital by private vehicle.

## DISCUSSION

Orthopedic injuries from snowmobile accidents have been documented previously.^1^ However, soft tissue injuries from snowmobile track, known as “shark bite” have not been previously described in the literature. Shark bite can be a major injury associated with snowmobiles or other recreational vehicles that rely on studded tracks for traction; emergency physicians in winter climates should be aware of this type of injury. While the repair of a laceration is commonplace for an emergency physician, consideration needs to be made for antibiotics and tetanus vaccination of these patients due to the nature of their injuries. Snowmobile tracks frequently contact soil and contaminated snow and could become broken and dislodged in these wounds. *Clostridium tetani* is a pathogen whose spores can survive in the soil during the winter.^2^

**CPC-EM Capsule**

What do we already know about this clinical entity?

*No similar information on the mechanism of this injury or images of the injury can be found in the literature. Standard wound care for lacerations is well known*.

What is the major impact of the image(s)?

*Bringing forth images not currently in the literature along with the unique name of “shark bite” that coincides with the injury and its mechanism*.

How might this improve emergency medicine practice?

*Providing physicians with these images provides knowledge of what to expect when encountering a snowmobile track laceration for those who do not often see them*.

## Figures and Tables

**Image 3:**
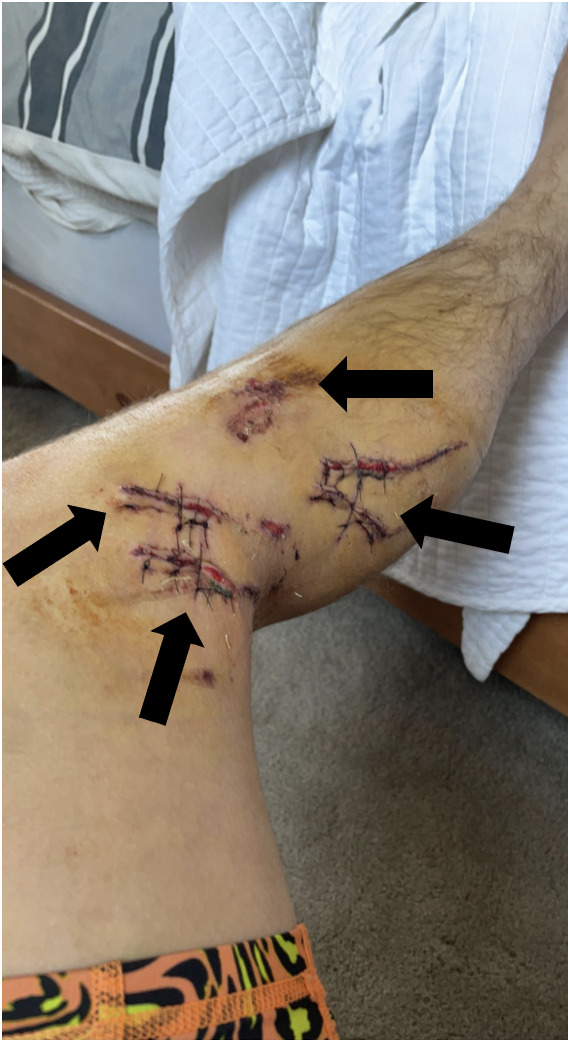
After washout and repair in the operating room, the leg in the image demonstrates closure of the lacerations (arrows) to the left lower extremity.
